# Implementing Pain Management Approaches Among Patients With Dementia in an Acute Hospital Setting: A Scoping Review

**DOI:** 10.1002/nop2.70529

**Published:** 2026-04-07

**Authors:** Faisal Mahama, Donna Brown, Deirdre Harkin, Vivien Coates

**Affiliations:** ^1^ Faculty of Life and Health Sciences Ulster University Londonderry UK; ^2^ Institute of Nursing and Health Research Ulster University Londonderry UK; ^3^ School of Nursing and Paramedic Science Ulster University Londonderry UK; ^4^ Ulster University Londonderry UK

## Abstract

**Aim:**

To determine which pain management approaches have been implemented in an acute hospital setting and gauge the extent to which they effectively assess and manage pain among patients with dementia.

**Design:**

The review followed the Joanna Briggs Institute framework, guided by the Preferred Reporting Items for Systematic Reviews and Meta‐Analysis extension for scoping reviews (PRISMA‐ScR).

**Method:**

The search strategy was designed to locate published and unpublished studies through online databases such as MEDLINE (Ovid) and CINAHL, as well as relevant grey literature sources, such as the WHO database. The review included primary and secondary research publications from 2005 to 2023 relating to patients with dementia in an acute hospital setting. The study focused on overall pain management approaches, including pain assessment, processes, and interventions to manage pain. Tables were used to extract and synthesise results using descriptive statistics.

**Results:**

Fourteen studies met the inclusion criteria. They included a range of designs: five cross‐sectional and two cohort studies; five interventional studies; one action research project; and one quality improvement programme. The interventions identified in the review mainly involved validating pre‐existing pain assessment tools, such as the Pain Assessment in Advanced Dementia Scale (PAINAD). Overall, the authors' primary strategy for implementing their respective interventions was organising participant awareness‐raising and training sessions.

**Conclusions:**

There is a lack of standardised, person‐centred protocols for managing pain in patients with dementia and a dearth of evidence relating to pain reduction among this client group. While valid pain assessment tools exist, their use alone does not guarantee timely or effective pain relief. Although studies reported the development of interventions to promote effective pain management, they did not consistently demonstrate a systematic reduction in pain among the patient groups. Whilst education on pain management is vital, it has been shown that education alone is insufficient to improve care.

**Implications for Practice:**

The review identified several validated instruments for assessing pain in people living with dementia; however, the availability of tools alone is not enough to change practice. A broader shift in pain management is required. Nurses need organisational support not only to incorporate observational pain assessment tools into routine workflows but also to connect assessments to clear clinical actions and adopt a proactive, holistic approach. This includes preventing diagnostic overshadowing by recognising pain as a potential cause of behavioural change and ensuring that pain management strategies go beyond assessment to deliver timely, person‐centred interventions. Health care providers need to support nursing staff beyond providing training and updates to encourage evidence‐based practice and to engender a culture in which pain management is a priority.

**Patient or Public Contribution:**

No patient or public contribution was made towards this review.

## 1 Introduction

Dementia is defined by WHO (2023, 1) as “a syndrome that can be caused by a number of diseases which over time destroy nerve cells and damage the brain, typically leading to deterioration in cognitive function (i.e., the ability to process thought) beyond what might be expected from the usual consequences of biological ageing”. It is considered a global health priority. However, even though there is extensive research on dementia, there is currently no cure or any effective prevention strategy (Mattia and Blasimme 2024). Therefore, quality nursing care has become an essential component in managing dementia. The risk of dementia escalates significantly with age, with an estimated 25%–30% of individuals aged 85 and above experiencing some degree of cognitive decline (WHO 2014). It is projected that the global number of people living with dementia will surge from 57.4 million in 2019 to 152.8 million in 2050 (Global Burden of Disease [GBD] 2022).

Due to the subjective nature of pain, it is difficult to measure it accurately and objectively (Wideman et al. 2019). Several studies have shown that people living with dementia regularly suffer from some degree of pain (Bullock et al. 2019; Jonsdottir and Gunnarsson 2021; van Kooten et al. 2015), with a growing amount of evidence revealing that pain is undertreated for people with dementia compared to matched controls (Hoffmann et al. 2014; Morrison and Siu 2000). Pain symptoms can be misinterpreted as behavioural symptoms of dementia (Sampson et al. 2015), leading to poor pain management in this population (Bonser 2025; Bullock et al. 2019). The gold standard for diagnosing pain is patient self‐report. However, it may be difficult for patients with dementia to self‐report their pain. It therefore requires healthcare team members to devise additional measures, such as using behavioural pain assessment tools or involving family members to help assess pain (Harmon et al. 2019).

Although there are more than 35 pain assessment tools, Peisah et al. (2014) highlighted several limitations. These include their complexity and the need for intensive observation, their lack of specificity for pain, reliance on unusual anchor points such as agitation or aggression, difficulty detecting pain in some individuals due to variability in pain expression, and the absence of clear guidelines on when to administer pain relief. Furthermore, pain assessment tools alone may be insufficient to change pain management practices (Brown and McCormack 2011). In addition to pain assessment tools, the WHO Pain Ladder serves as a decision support tool to assist in recognising and managing pain in patients with dementia. It is, however, imperative to note that they are not routinely used in practice nor implemented within a decision‐theory framework (Lichtner et al. 2016). Pain assessment for people with dementia is, therefore, challenging and complex. There is a need to develop and implement a pain management approach that has the potential to manage pain effectively among patients with dementia. Untreated pain in people with dementia has severe implications for quality of life. Pain is associated with the presence, onset or exacerbation of depression, delirium, sleep disturbance, cognitive decline, resistive behaviour, and neuropsychiatric symptoms (Ahn et al. 2015; Pieper et al. 2013). Although it is challenging to manage pain in patients with dementia, there is an ongoing collaborative effort to develop pain management approaches for people with dementia (Bullock et al. 2020; Harkin et al. 2022).

People living with dementia often experience both acute and chronic pain, yet managing that pain in acute care settings remains a significant challenge (Smith et al. 2023). Communication difficulties, cognitive impairment, and the fast‐paced nature of hospital environments frequently lead to under‐recognition and undertreatment of all forms of pain in an acute care setting (Pu et al. 2025). Furthermore, sub‐optimal pain management practices among patients with dementia in an acute hospital setting are influenced by the context in which health personnel work, their shared way of thinking and working, and how sources of evidence are used (Harkin et al. 2022). A holistic approach, one that addresses both immediate and long‐term pain needs, is essential but is rarely implemented consistently for this population (Liao et al. 2023; Resnick et al. 2023). Improving pain management strategies in acute care is therefore critical to ensure dignity, comfort, and better clinical outcomes for people living with dementia (Ingelson et al. 2024). Harkin et al. (2022) have identified the need for holistic person‐centred approaches to be part of every interaction with people with dementia if pain management is to be improved. Working with key stakeholders, an innovative tool (DOTS) has been developed to assist multidisciplinary teams in seeing the person as a whole when assessing the pain of patients affected with dementia. Exploring holistic pain management approaches in acute care led to a preliminary search of three databases (CINAHL, PubMed, and ETHOS), which found no scoping review on the above‐stated topic. Therefore, this review aims to determine which pain management approaches have been implemented in an acute hospital setting and gauge the extent to which they assess and manage pain effectively among patients with dementia.

## 2 Aim

This scoping review aimed to determine which pain management approaches have been implemented in an acute hospital setting and gauge the extent to which they effectively assess and manage pain among patients with dementia.

## 3 Methods

### 3.1 Study Design

The review adapted the framework proposed by the Joanna Briggs Institute (JBI) (Peters et al. 2020). The Preferred Reporting Items for Systematic Reviews and Meta‐Analysis extension for scoping reviews (PRISMA‐ScR) (Tricco et al. 2018) was used to guide the reporting of the scoping review (see Table S1). The authors developed an a priori protocol that defined the population, concept, and context (PCC) for the study, the data sources, the search strategy, the inclusion/exclusion criteria, and the procedure for screening articles, extracting, and analysing the data. This protocol was subsequently registered with the Open Science Framework (Registration: DOI https://doi.org/10.17605/OSF.IO/EBFSU) before the commencement of the study.

### 3.2 Definitions of Population, Concept and Context (PCC)

The populations of interest in this review were people living with dementia and healthcare staff who care for these people in hospital settings.

The study focused on pain management approaches as the primary concept. In this review, the term ‘pain’ is used inclusively to refer to both acute and chronic pain among people living with dementia, recognising that chronic pain arising from conditions such as osteoarthritis remains highly prevalent and influences pain presentation in acute care settings (Achterberg et al. 2021). Although the review considered all types of pain, the focus was mainly on acute care, as evidence indicates that pain management in this area is often inadequate. Published and unpublished studies conducted from 2005 to 2023 were considered. Studies where access to full‐text articles or documents could not be obtained were excluded. The review also identified the outcomes of pain management approaches.

The study's context was acute hospital settings, where patients received active but short‐term treatment. The acute hospital settings included in this study were the surgical and medical units, intensive care units, emergency departments, geriatric units, and orthopaedic units. Nursing homes, care homes, and residential homes were excluded from the study.

### 3.3 Review Question

What pain management approaches have been implemented, and what evidence exists regarding their effectiveness for managing pain for those with dementia in acute hospital settings? The research question was developed using the PCC framework.

### 3.4 Inclusion and Exclusion Criteria

This study's inclusion and exclusion criteria were strictly based on the PCC framework and the review question/objective (Table 1). The authors scrutinised studies to ensure that all the established criteria were met before including them in the review. Due to a lack of translation capacity, the authors only considered studies published in English during title and abstract screening.

**TABLE 1:** Inclusion and exclusion criteria.

Study element	Inclusion	Exclusion
Population	The study involved patients with dementia and/or healthcare staff	Other cognitive impairments or neurological disorders, such as epilepsy and delirium
Concept	Pain assessment and management Written in English with full text freely available Published between 2005 and 2023. Primary/secondary research/Grey literature The reported study includes an intervention	Palliative care End‐of‐life care
Context	The study was conducted in an acute and/or sub‐acute care setting Hospital	Nursing homes, care homes and residential homes Long‐term care

### 3.5 Types of Sources

The review considered primary research studies across quantitative, qualitative, and mixed‐method designs. Experimental and quasi‐experimental study designs, including randomised controlled trials, non‐randomised controlled trials, before‐and‐after studies, and interrupted time‐series studies. Additionally, analytical observational studies and descriptive observational study designs were considered for inclusion. Qualitative studies based on approaches such as phenomenology, grounded theory, ethnography, qualitative description, action research, and feminist research were included. Systematic reviews that met the inclusion criteria were also screened depending on the research question. Government papers, PhD theses, editorials, and reports (such as from quality improvement studies) were also potentially relevant for inclusion in this scoping review.

### 3.6 Search Strategy

The database search for this scoping review was conducted between 20 March 2023 and 5 July 2023. However, alerts were set up across all databases for 1 year to notify the research team of new publications on the review topic. It is essential to note that by 5 July 2024, no new publications on the review topic were identified. The development and implementation of the search strategy were achieved with the assistance of a subject specialist librarian. It was aimed at locating both published and unpublished studies. The following databases were searched in a stepwise manner: MEDLINE (Ovid), EMBASE, Cumulative Index to Nursing and Allied Health Literature (CINAHL), and ProQuest Health and Medical Collection. The PCC framework served as a guide for modifying the search strategy. Key terms and phrases were determined, and each database's search terms were optimised (see Figure S2).

The first author also hand‐searched the reference lists of included studies to identify additional relevant studies. A search of relevant grey literature sources (WHO database, Grey Literature Report, OpenGrey, and Web of Science Conference Proceedings) was conducted to ensure that all relevant information was captured for this study. The search strategy included all dissemination languages. Papers and other documents not written in English were noted to provide a sense of the overall available literature. However, these papers were not considered for full review due to a lack of translation capacity. In total, eleven non‐English papers were excluded during the screening of titles and abstracts.

The research team's decision to only consider studies conducted from 2005 was borne out of the fact that, although activity in Implementation Science started in the 1990s (Cook et al. 2017; Claridge and Fabian 2005), it became much more prominent and well‐defined in 2005 (Boulton et al. 2020). In addition, considering the substantial changes in healthcare, pharmacology, and documentation over the past 20 years, it was assumed that the papers published before 2005 would be less applicable to today's health services.

The development and implementation of the search strategy were achieved as follows:

1. An initial search of all the databases was undertaken to identify articles on the review topic.
2. The second search used the search strategy in the specified grey literature locations, including all identified keywords and index terms.
3. Reference list search: The reference lists of included articles were searched for additional relevant articles.
4. Alerts were set up on all the databases to inform the research team about the publication of new studies related to the review topic.

### 3.7 Study/Source of Evidence Selection

**FIGURE 1:**
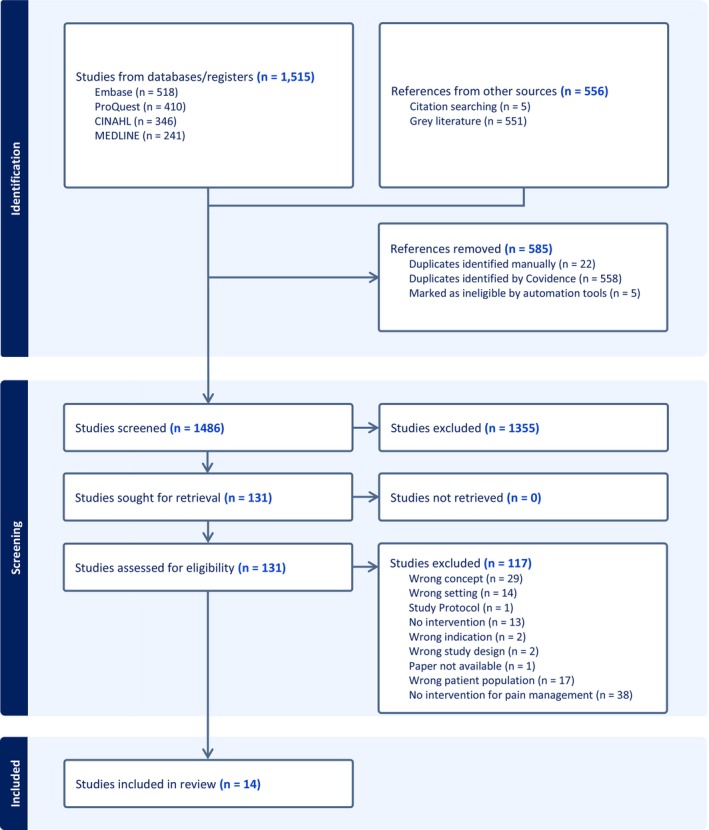
PRISMA flowchart.

The PRISMA (2020) flow diagram for new systematic reviews (Page et al. 2021) was used as a guideline for the source of evidence selection (Figure 1).

After completing the search, all identified citations were collated in RefWorks and uploaded into Covidence (Covidence Systematic Review Software 2024), and duplicates were identified and removed.

Three authors conducted a pilot test of source selectors before commencing source selection across the team. This enabled the team to refine the source selection tool. During the pilot, the authors randomly selected and independently screened 25 titles and abstracts using the inclusion and exclusion criteria. The team then met to discuss discrepancies and modify the inclusion and exclusion criteria. Three authors commenced full‐scale screening of titles and abstracts for possible eligibility when at least 75% agreement was achieved. After screening titles and abstracts, eligible studies were added to the full‐text review.

All three authors independently assessed the full text of selected citations in detail against the inclusion and exclusion criteria and the review's objective. Reasons for excluding sources of evidence in full text that do not meet the inclusion criteria were recorded and reported in the scoping review. Any conflicts at each stage of the selection process were resolved through discussion or by involving an additional reviewer.

### 3.8 Data Extraction

The authors developed a data extraction tool, adapted from the Joanna Briggs Institute's data extraction template (Peters et al. 2020), to confirm study relevance and extract study characteristics. The first author extracted data from papers included in the scoping review via Covidence (Covidence Systematic Review Software 2024). The extracted data included specific details on the participants, the concept, the context, the study methods, and the key findings relevant to the review question. The draft data extraction tool was modified and revised as needed during data extraction from each included evidence source. The three other authors subsequently reviewed the extracted data to ensure it reflected each associated study's characteristics. Any conflicts were resolved through discussion or with additional reviewers.

### 3.9 Data Analysis

The primary objective of a scoping review is to provide an overview of available data. Arksey and O'Malley (2005) indicated that a scoping review requires an analytical framework or a thematic construction to provide a narrative account of available literature. Extracted data were tabulated and collated according to evidence source details and characteristics of included studies, as well as details/results extracted from the evidence sources (Tables 3, 4, 5, 6). A descriptive‐analytical method was adopted to summarise and synthesise the results accordingly. Subsequently, the charted qualitative data were presented narratively. Data synthesis included the study characteristics such as study design, conceptual/theoretical framework, participants, concept, context, results, type of pain management approach, and outcome variables. The Patterns, Advances, Gaps, Evidence for Practice and Research (PAGER) framework developed by Bradbury‐Jones et al. (2021) was used to analyse and comprehensively describe the included studies (see Table 2).

**TABLE 2:** Summary of analysis using the PAGER framework.

Patterns	Advances	Gaps	Evidence for practice	Research recommendations
Studies testing for the validity and or reliability of pain assessment tools	Pain assessment tools such as Pain Assessment in Advanced Dementia (PAINAD) are valid and reliable in effectively assessing and managing pain in patients with dementia	The use of a valid and reliable pain assessment tool alone is not adequate to bring about pain reduction in persons living with dementia	It is recommended that healthcare staff use observational/behavioural pain assessment tools for people with dementia due to their ability to improve pain recognition and intensity measurement	Repeated studies are needed to build a body of work on the validity and reliability of these pain assessment tools
The development and implementation of pain management approaches	It is important to develop innovative pain management approaches to improve pain management in patients with dementia	There is a lack of standardised, person‐centred protocols for managing pain in patients with dementia	A person‐centred approach that requires healthcare staff to develop a clear understanding of the background of the patient with dementia, their interests, and personal history should be adopted to improve pain management	Research should focus on developing holistic person‐centred pain management approaches that consider the patient with dementia as a whole when managing pain
Strategies to successfully implement pain management approaches	Implementation strategies, including training, in‐service meetings, and clinical supervision, appear essential when implementing an intervention	Insufficient knowledge of dementia and inadequate training hindered implementation; thus, education and training were required to ensure successful implementation	Training sessions should be organised and delivered in clinical settings to enhance healthcare staff's practice	Recommends future studies to explore the barriers and facilitators to implementing a pain management approach in patients with dementia successfully

## 4 Results

### 4.1 Overview of the Search Strategy

The search strategy produced 1515 potentially relevant studies from the four databases (Embase = 518, ProQuest = 410, CINAHL = 346 and MEDLINE = 241). A total of 556 references from other sources were retrieved. This included 551 articles from a grey literature search and 5 from manual searches for relevant references. Duplicate references were removed (*n* = 580). Duplicates identified manually were 22, whilst Covidence automatically identified and removed 558. After the duplicates and 5 references marked as ineligible were removed, 1486 studies were screened by title and abstract, and 131 were sought for retrieval. Of the 131 studies, 117 were excluded, leaving 14 for inclusion in the review. The authors have provided a detailed description of the search strategy's outcomes using the PRISMA flowchart (Figure 1).

### 4.2 Evidence Source Details and Characteristics of Included Studies

#### 4.2.1 Year, Country, and Publication

The 14 studies included in this scoping review were published between 2005 and 2022 (Tables 3 and 4). Eight studies were published between 2018 and 2022. Most publications originated from Europe (*n* = 10); UK = 5, Italy = 2, Germany = 1, Spain = 1 and Switzerland = 1. This was followed by the United States of America (*n* = 2) and Australia (*n* = 2). All the included studies were published in peer‐reviewed academic journals.

**TABLE 3:** Evidence source details and characteristics (studies testing the validity, reliability or feasibility of a pain assessment tool).

Study ID Author Year Country	Title	Publication	Objective/Aim of the study	Study design	Population description (sample size)	Study setting	Data collection method	Intervention	Implementation strategies	Data analysis
1. Costardi et al. 2007 Italy	The Italian version of the pain assessment in advanced dementia (PAINAD) scale	*Archives of Gerontology and Geriatrics*, 44(2), 175–180	The purpose of the present study was to assess the validity of the PAINAD in the Italian version as a reliable tool for measuring pain in demented people	Cross‐sectional study	Patients with dementia (20)	Geriatric Evaluation and Rehabilitation Unit (GERU), Richiedei Medical Center, Palazzolo, Northern Italy	Observation	PAINAD	An expert rater administered the intervention. The PAINAD was translated by a committee of two physicians and a psychologist, and the back translation by a native‐speaking English teacher	SPSS 8.0 for Windows Descriptive statistics Inferential statistics
2. Fry et al. 2018 Australia	Can an observational pain assessment tool improve time to analgesia for cognitively impaired older persons? A cluster randomised controlled trial	*Emergency Medicine Journal: EMJ*, 35(1), 33–38	To measure the impact of an observational pain assessment dementia tool on time from ED arrival to the first dose of analgesic medicine	Randomised controlled trial	Patients with cognitive impairment and bone fracture (602)	Eight metropolitan EDs in Sydney, Australia	Observation Manual examination of the patient's medical and e‐Health records	PAINAD	Audits of participating sites before the commencement of the study. Delivery of an education programme across all sites before the trial. The organisation of regular in‐service meetings to facilitate education and study engagement. Non‐intervention sites: Pain assessment was performed according to usual care. Intervention sites: A SIS score of 4 or less requires the nurse to use the PAINAD to assess pain	Descriptive statistics Inferential statistics Statistical analysis was conducted using IBM SPSS V.21
3. Pautex et al. 2005 Switzerland	Feasibility and reliability of four pain self‐assessment scales and correlation with an observational rating scale in hospitalised elderly demented patients	*The Journal of Gerontology. Series A, Biological Sciences and Medical Sciences*, 60(4), 524–529	To evaluate the feasibility and reliability of four pain self‐assessment scales in this population and compare their performance to an observational pain rating scale	Prospective clinical study	Patients with dementia (160)	Geneva University Geriatric Hospital	Questionnaire Observation	The horizontal visual analogue scale (HVAS) The vertical visual analogue scale (VVAS) The faces pain scale (FPS) The 6‐point verbal rating scale (VRS)	Patients were asked if they experienced any pain at the time of the assessment. Patients were asked to point to the adjective that best describes their current pain. The examination was completed in a quiet room with the patient in a sitting position, under the supervision of the examiners. An observational pain assessment scale, Doloplus, was completed independently by the nursing staff in charge of the patient	Analyses were performed with the Stata 7.0 statistical package and SPSS release 11.0 Descriptive statistics Inferential statistics
4. van de Rijt et al. 2019 UK	Orofacial pain during rest and chewing in people with dementia admitted to acute hospital wards: validity testing of the orofacial pain scale for non‐verbal individuals	*Journal of Oral & Facial Pain and Headache*, 33(3), 247–253	To assess the validity of the components resting and chewing of the recently developed observational diagnostic tool, the Orofacial Pain Scale for Non‐Verbal Individuals (OPS‐NVI)	Cross‐sectional observational study	Patients with dementia (56)	Two UK hospitals	Observation Questionnaires	The orofacial pain scale for non‐verbal individuals (OPS‐NVI)	Participants were observed for 3 min during rest and for 3 min while eating a routine meal or snack. Participants were asked whether they experienced pain in the orofacial area during each activity after the OPS‐NVI observation. The Numeric Rating Scale (NRS), the Verbal Descriptor Scale (VDS), and the Faces Pain Scale‐Revised (FPS‐R) were used in case pain was present during the activity	SPSS Version 24 Software was used for data analysis. Descriptive statistics Inferential statistics
5. Oosterman et al. 2014 UK	When pain memories are lost: a pilot study of semantic knowledge of pain in dementia	*Pain Medicine (Malden, Mass.)*, 15(5), 751–757	To explore the semantic concepts of pain in people with dementia and whether this is associated with clinical pain report	Cohort study with nested cross‐sectional analysis	Older people with dementia (39)	Acute general hospital medical wards for older people	Observation	Semantic memory for pain	Patients with dementia were first asked whether they were in pain now and then indicated their current pain intensity using the Wong‐Baker FACES Pain Rating Scale (FACES). Pain was assessed using the Pain Assessment in Advanced Dementia (PAINAD) scale	Data were analysed using SPSS 19.0. Descriptive statistics Inferential statistics
6. Natavio et al. 2020 USA	A comparison of the pain assessment checklist for seniors with limited ability to communicate (PACSLAC) and pain assessment in advanced dementia scale (PAINAD)	*Pain Management Nursing: Official Journal of the American Society of Pain Management Nurses*, 21(6), 502–509	To determine interrater reliability of the PACSLAC and PAINAD in assessing pain behaviours in patients with the same pain stimulus. To determine the consistency of the reliable changes between and within the two instruments at separate time points. To assess the preferences of nurses using both the PACSLAC and PAINAD	A single‐group, within‐subjects repeated‐measures design	Patients with dementia (30). Nurses (20)	Orthopaedic unit	Observation Survey	Pain assessment checklist for seniors with limited ability to communicate (PACSLAC). Pain assessment in advanced dementia scale (PAINAD)	Participants were assessed at three time points (24, 48, and 72 h) in the first 72 h after surgery. Pre‐study training of nurses. Provision of visual instructional aides to each nurse for reference	Data analysis was conducted using IBM SPSS Statistics software (version 25). Descriptive statistics. Inferential statistics
7. Ferrari et al. 2009 Italy	Pain assessment in non‐communicative patients: The Italian version of the non‐communicative patient's pain assessment instrument (NOPPAIN)	*Ageing Clinical and Experimental Research*, 21(4–5), 298–306	To verify if the Italian version of the non‐communicative patients' pain assessment instrument (NOPPAIN) could be used in a hospital setting	Randomised controlled trial	Nursing staff members (45) Patients with severe dementia (Treatment Group) (60) Cognitively intact patients (Control Group) (42)	The General Medicine and Geriatric wards of Vicenza Hospital	Observation	NOPPAIN	Nursing staff members undertook a brief standardised training programme for the use of NOPPAIN. Pain assessments were performed after care activities in the morning and before analgesic administration	Statistical package for social sciences, version 14.0 for Windows. Descriptive statistics Inferential statistics
8. Sampson et al. 2015 UK	Pain, agitation, and behavioural problems in people with dementia admitted to general hospital wards: a longitudinal cohort study	*Pain*, 156(4), 675–683	To investigate the prevalence of pain in people with dementia admitted to general hospitals and explore the association between pain and behavioural and psychiatric symptoms of dementia (BPSD)	Longitudinal cohort study	Patients with dementia (230)	Two large acute general hospitals in London	Observation Discussion Hospital notes	The Pain Assessment in Advanced Dementia Scale (PAINAD) Wong‐Baker FACES scale The Cohen‐Mansfield Agitation Inventory (CMAI) The Behavioural Pathology in Alzheimer's Disease Scale (BEHAVE‐AD)	Assessors received training in the use of all tools. PAINAD was rated first. Pain was observed at rest and during movement. Participants were asked whether they were in pain, then shown the Wong‐Baker FACES scale. A different research assistant assessed agitation using the Cohen‐Mansfield Agitation Inventory (CMAI). The authors used the Behavioural Pathology in Alzheimer's Disease Scale (BEHAVE‐AD) Data were collected at study entry and then every 4 (±1) days for pain and BPSD	Descriptive statistics Inferential statistics
9. Dunford et al. 2022 UK	Psychometric evaluation of the pain assessment in advanced dementia scale in an acute general hospital setting	*International Journal of Geriatric Psychiatry*, 37(12), 10.1002/gps.5830	To evaluate the PAINAD by investigating its psychometric properties in people with dementia in acute general hospitals	Cross‐sectional study	Patients with dementia (230)	Two National Health Service (NHS) acute general hospitals in London	Observation Discussion Case Notes	PAINAD	Training of researchers. Pain assessment was carried out at rest and during movement. Pain was also assessed with the Wong‐Baker FACES scale. The authors measured behavioural and psychological symptoms of dementia using the Behavioural Pathology in Alzheimer's Disease Scale (BEHAVE‐AD)	Descriptive statistics Inferential statistics

**TABLE 4:** Evidence source details and characteristics (studies that developed and implemented a pain management approach).

Study ID Author Year Country	Title	Publication	Objective/Aim of the study	Study design	Population description (sample size)	Study setting	Data collection method	Intervention	Implementation strategies	Data analysis
10. Graham et al. 2022 Australia	Hospital nurses' management of agitation in older cognitively impaired patients: Do they recognise pain‐related agitation?	*Age and Ageing*, 51(7), afac140	To investigate hospital nurses' management of agitation in older cognitively impaired patients with pain	Descriptive Correlational Study	Registered medical and surgical nurses (274)	Ten public hospitals in Queensland, Australia	Observation Questionnaire	Innovative virtual simulation of a vignette	Simulation training video. Interaction with the avatar	Descriptive statistics Inferential statistics Conceptual content analysis
11. Gilmore‐Bykovskyi et al. 2021 USA	Implementation and Evaluation of an Acute Care Multicomponent Intervention for Dementia‐Related Behavioural Expressions	*Journal of Gerontological Nursing*, 47(9), 21–30	To determine the effect of the Personalised Approach and Targeted Interventions (PROACTIVE) Treatment Approach on nursing staff stress and confidence in relation to dementia‐related behavioural expressions (BE) management. To determine the feasibility and utility of the intervention components	Quality Improvement Program	Patients with cognitive impairment due to dementia. (40)	A 20‐bed general medicine inpatient unit in a Midwestern Veterans Affairs Hospital, USA	Observation Discussion Survey Data abstraction from the EHR	The Personalised Approach and Targeted Interventions (PROACTIVE) Treatment Approach	PROACTIVE was developed in collaboration with nursing leadership and nursing staff working in the intervention unit. Training of nurses through a 2‐h in‐service incorporating didactic and hands‐on training. Regular bi‐weekly meetings to discuss barriers to implementation	Descriptive statistics Inferential statistics Qualitative content analysis
12. Lukas et al. 2022 Germany	Responsive behaviours and pain management in hospital dementia care: A before and after comparison of the serial trial intervention	*Frontiers in Pain Research (Lausanne, Switzerland)*, 3, 810804	To reduce the level of behavioural and psychological symptoms of dementia (BPSD) in an acute hospital environment through a stepwise procedure followed by the initiation of a needs‐oriented treatment	Open, prospective, interventional study	Patients with dementia (107)	A general hospital in Germany	Observation Questionnaires	Serial Trial Intervention (STI)	Scientific experts in STI and pain supported the development of a curriculum. Conduction of a trainee programme based on STI and including pain assessment. Mentor training sessions Before implementation Group: Unstructured pain assessment using NRS and VRS After Implementation Group: Structured pain assessment using the modified STI	Descriptive Analysis Inferential Statistics All analyses were performed using IBM SPSS Statistics for Windows version 26.0
13. McCorkell et al. 2017 UK	Care of patients with dementia in an acute trauma and orthopaedics unit	*Nursing Standard (Royal College of Nursing (Great Britain): 1987)*, 31(36), 44–53	To increase awareness of the needs of patients with dementia in the trauma and orthopaedics unit of one acute hospital, and to collaborate with staff on the unit to identify ways of improving the care experienced by these patients and their families	Action research approach	Patients with dementia who are receiving orthopaedic treatment (20) Staff Nurses (2)	Trauma and orthopaedics unit, Altnagelvin Hospital, Londonderry	Observation Audits	Dementia Toolkit	Clinical supervision session. Audits of the care of patients for baseline data. Meeting with nursing staff. Development of dementia toolkit. Change the colour of patients with dementia folders from black to purple. Teaching and training sessions. Selection of dementia champions	Descriptive statistics
14. Montoro‐Lorite et al. 2020 Spain	Integrated management of pain in advanced dementia	*Pain Management Nursing: Official Journal of the American Society of Pain Management Nurses*, 21(4), 331–338	To develop and evaluate the implementation of a protocol for the comprehensive management of pain in advanced dementia	Quasi‐experimental study	Patients with dementia (22)	Acute Geriatric Unit (AGU) in a high‐technology hospital in Barcelona	Observation	Comprehensive Pain Management Protocol in Advanced Dementia	Training of nurses. Intervention Group: Consisted of patients with odd patient identification numbers. Perception of pain was assessed using the PAINAD‐Sp scale and a nurse's report based on the NRS scale. Control Group: Consisted of patients with an even patient identification number Perception of pain was assessed through a nurse's report using only the NRS scale. Pain assessments were carried out five times a day: two during rest (after meals), two during painful activity (physical movements), and one at night during rest	A univariate analysis was carried out to determine the frequency and distribution of the variables using SPSS Statistics Base 22.0. Descriptive statistics Inferential statistics

#### 4.2.2 Study Design and Objective

From Tables 3 and 4, half of the included studies were observational studies (Costardi et al. 2007; Dunford et al. 2022; Graham et al. 2022; Oosterman et al. 2014; Pautex et al. 2005; Sampson et al. 2015; van de Rijt et al. 2019). In addition, five studies (Ferrari et al. 2009; Fry et al. 2018; Lukas et al. 2022; Montoro‐Lorite et al. 2020; Natavio et al. 2020) were identified as Interventional Studies. The other design types used were Action Research (McCorkell et al. 2017) and a Quality Improvement Programme (Gilmore‐Bykovskyi et al. 2021). The overall objective of most studies was to either validate a pain assessment tool (*n* = 6) (Costardi et al. 2007; Dunford et al. 2022; Ferrari et al. 2009; Fry et al. 2018; Natavio et al. 2020; van de Rijt et al. 2019) or test the feasibility and reliability of pain assessment tools (*n* = 1) (Pautex et al. 2005). Five studies (Graham et al. 2022; Gilmore‐Bykovskyi et al. 2021; Lukas et al. 2022; McCorkell et al. 2017; Montoro‐Lorite et al. 2020) focused on developing and implementing pain management approaches to effectively assess and manage pain in patients with dementia. The purpose of the two remaining studies (Oosterman et al. 2014; Sampson et al. 2015) was to create awareness of dementia and establish measures for effectively assessing and managing pain in patients with dementia.

#### 4.2.3 Population Description and Study Setting

The participants in the included studies were patients with dementia (*n* = 9) and patients with dementia who had been treated for other conditions, such as bone fractures (*n* = 3) (Tables 3 and 4). Four studies recruited both nurses and patients with dementia. However, in one study, only nurses were recruited. In another study, the participants included nurses, patients with dementia (intervention group), and cognitively intact patients (control group). The settings were categorised into three types: Acute General Hospital (*n* = 8), Geriatric Unit/Hospital (*n* = 4), and Trauma/Orthopaedic Unit (*n* = 2).

#### 4.2.4 Intervention and Implementation Strategies

A range of interventions were identified in the included studies (Tables 3 and 4). Most studies (*n* = 9) (Costardi et al. 2007; Dunford et al. 2022; Ferrari et al. 2009; Fry et al. 2018; Natavio et al. 2020; Oosterman et al. 2014; Pautex et al. 2005; Sampson et al. 2015; van de Rijt et al. 2019) used existing interventions (Table 3). These interventions were PAINAD, HVAS, VVAS, FPS, VRS, OPS‐NVI, PACSLAC, NOPPAIN, CMAI, BEHAVE‐AD, and semantic memory for pain. The other five studies (Gilmore‐Bykovskyi et al. 2021; Graham et al. 2022; Lukas et al. 2022; McCorkell et al. 2017; Montoro‐Lorite et al. 2020) developed, implemented, or evaluated a new intervention (Table 4). These new interventions covered a wide range of actions: virtual stimulation of a vignette, PROACTIVE, STI, dementia toolkit, and a comprehensive pain management protocol in advanced dementia. The interventions involved a variety of components. The most frequent one was the organisation of participant training sessions (*n* = 10). Experts developed or administered two of the interventions (Costardi et al. 2007; Lukas et al. 2022). In one study (Costardi et al. 2007), the pain assessment scale was translated into English, whereas in another study (Pautex et al. 2005), the assessment scale was independently implemented in a conducive environment. Two studies employed audits (Fry et al. 2018; McCorkell et al. 2017) and one conducted clinical supervision for participants (McCorkell et al. 2017).

#### 4.2.5 Data Collection Method and Analysis

Observation was the most common data‐collection method across all studies (Tables 3 and 4). This was followed by data abstraction from Electronic Health Records (EHR) and patients' notes (*n* = 5), questionnaires (*n* = 4), discussions (*n* = 3), and surveys (*n* = 2). All the studies analysed quantitative data using descriptive or inferential statistics through various versions of SPSS for Windows. However, qualitative data were analysed by qualitative content analysis (*n* = 2).

#### 4.2.6 Theoretical/Conceptual Framework

Only three studies (Tables 3 and 4) reported using a theoretical or conceptual framework: the Conceptual Model of the Influences on Nurses' Clinical Performance underpinned by a Dual‐Processing Framework (DPT) (Graham et al. 2022), Replicating Effective Programs Implementation Model (Gilmore‐Bykovskyi et al. 2021), and Snow's Conceptual Model of Pain Assessment for Non‐Communicative People with Dementia (Natavio et al. 2020).

### 4.3 Details/Results Extracted From the Source of Evidence (About the Concept of the Scoping Review)

#### 4.3.1 Analysis Approach of the Implemented Intervention

The main statistical approaches used in the studies were parametric and non‐parametric tests (*n* = 10) (Tables 5 and 6). The most common examples include the Mann–Whitney *U* test, the Chi‐square test, the Spearman's rho correlation coefficient, and Fisher's exact test. The other evaluation approaches were as follows: before and after comparison (Lukas et al. 2022), post‐toolkit audit (McCorkell et al. 2017), post‐simulation questionnaire (Graham et al. 2022), exit survey (Gilmore‐Bykovskyi et al. 2021; Natavio et al. 2020), and retrospective charts/notes review (Gilmore‐Bykovskyi et al. 2021).

**TABLE 5:** Details/Results extracted from studies that tested for validity, reliability, or feasibility of pain assessment tools.

SN	Study ID: Author Year Country	Evaluation approach	Most relevant finding(s)	Barrier(s)
1	Costardi et al. 2007 Italy	The Visual Rating Scale (VRS) evaluated pain and measured the concurrent validity of the PAINAD	The PAINAD does not imply cognition, abstract reasoning, or verbal skills, which are damaged in cognitively impaired older adults PAINAD can be easily administered after appropriate training	The sample group was small (*n* = 20). Pain was measured at only one point in time PAINAD was subject to rater distortion when raters were not well‐trained
2	Fry et al. 2018 Australia	The Mann–Whitney *U* test was used to determine the primary outcome Pearson's *χ* ^2^ test was used to determine differences in proportions for secondary outcomes A Cox regression analysis was used to measure the time of analgesia Binary logistic regression was used to estimate the proportion of patients who received pain medication within 60 min	Implementing an evidence‐based pain assessment tool is complex. EDs may require individualised multifactorial implementation considerations, including cultural, transdisciplinary, and human factors, to improve pain management for people with cognitive impairment	Missing data in a small number of patients. Nurses screening suspected long‐bone fractures using the SIS Cognitive impairment could not be independently verified in over half of the patients The study did not determine the appropriateness of the analgesic medication
3	Pautex et al. 2005 Switzerland	Categorical variables were evaluated by chi‐square or Fisher's exact test The intraclass correlation coefficient (ICC) was used to measure inter‐rater and inter‐rater reliability Spearman's rho correlation coefficient was used to assess the strength of association between pain intensity scores across the different scales The Wilcoxon matched‐pairs signed‐rank test was used to evaluate whether the observational scale under‐ or overestimated pain compared to self‐assessment	Completing an assessment does not imply reliability. This is a key issue because unreliable measurements cannot be used effectively to detect pain or, more importantly, to measure change	The use of a rigorous definition of scale comprehension The study involved a few patients with severe dementia, and they may not have been able to determine the differences between the four scales due to severe memory loss
4	van de Rijt et al. 2019 UK	The concurrent validity of the OPS‐NVI was assessed by using Spearman's coefficient, with a significance level of *p* < 0.05 The sensitivity, specificity, and the Area Under the Receiver Operating Curve (AUROC) were calculated for each activity by comparing the presence of orofacial pain according to the OPS‐NVI, with the presence of pain according to self‐report The Spearman's coefficient, with a significance level of *p* < 0.05, was used to determine if the single behaviour items and the total number of ‘yes’‐scored behaviour items with the OPS‐NVI are related to the presence of orofacial pain or according to self‐report The prevalence‐adjusted and bias‐adjusted kappa (PABAK) was used to determine if the presence of orofacial pain according to the OPS‐NVI agrees with the presence of orofacial pain, according to self‐report To identify the size of the correlations, Cohen's guidelines were used	Participants who self‐reported the presence of orofacial pain were likely to have more observed pain‐indicative behaviour	Small sample size (*n* = 56) Only the OPS‐NVI components ‘resting’ and ‘chewing’ were used One researcher collected all the data
5	Oosterman et al. 2014 UK	Mann–Whitney *U* tests were performed to examine whether semantic memory for pain diminished in dementia Potential group differences in these different categories were examined using Pearson chi‐square tests Spearman rank correlations were calculated to examine the relationship between general cognition (MMSE), semantic memory for pain (SMP test), pain observations (PAINAD), and between the SMP test and clinical pain report	The ability of patients with dementia to use the FACES scale positively correlated with the number of pain pictures correctly identified	The findings of an association between semantic memory and clinical pain reports were based on correlation, and no causal relationship can be assumed Small sample size of patients with dementia (*n* = 26) The researcher was not blinded to the participant's status
6	Natavio et al. 2020 USA	Intraclass correlations (ICC) based on consistency were used to measure inter‐rater reliability Reliable change values were used to evaluate the differences in pain scores over the three time periods assessed The end‐of‐study nurse survey (exit survey) provided demographic data on nurses and tool preferences	The level of dementia beyond a certain degree does not affect the assessment of pain, but the medication dosage at a certain point may	Small sample size (*n* = 30) Participant selection took place at one hospital The burden of using both tools for assessment
7	Ferrari et al. 2009 Italy	Inter‐rater correlation coefficients were computed to determine the extent of agreement between the two raters' NOPPAIN ratings The correlation between the NOPPAIN SCORE and OERS scores for the two patient groups was computed to examine the relationship between behavioural expressions of pain and affective states The possibility of using NOPPAIN in a hospital setting was examined by considering the time needed to compile the NOPPAIN form	The findings stressed the importance of using structured assessment tools to guide nursing staff in focusing on specific pain behavioural indicators	Small sample size (*n* = 60) Patients with dementia were significantly more compromised than control patients in the level of daily functioning
8	Sampson et al. 2015 UK	Generalised estimating equations (GEE) were used to compute prevalence and 95% confidence intervals over all assessments during the admission The authors tested for associations between demographic and clinical characteristics of the participants and the presence of pain during admission using *χ* ^2^ or Fisher's exact test, as appropriate They also examined the association between the PAINAD score and the total CMAI and the BEHAVE‐AD total scores using GEEs with exchangeable correlation structures and robust standard errors A sensitivity analysis was conducted due to potential overlap in the symptoms used by both scales	Only older age was associated with an increased prevalence of self‐reported and observed pain (on movement and at rest) Those admitted with “falls, fracture, or pain” or “cardiac” events had lower pain prevalence	Recall bias may have led to over‐reporting of “troublesome” behaviours Residual confounding may have occurred
9	Dunford et al. 2022 UK	Concurrent validity was assessed by collecting FACES measurements at the same time as PAINAD at rest Convergent validity was evaluated by the same researcher, who applied BEHAVE‐AD simultaneously with PAINAD at rest and during activity Discriminant validity was determined by comparing PAINAD scores at rest and during activity using the Wilcoxon signed‐rank test Cronbach's alpha coefficient was used to analyse internal consistency at rest and during activity Inter‐rater reliability was assessed by two trained researchers independently applying the PAINAD, during activity and at rest, to one participant simultaneously	The authors found that PAINAD could discriminate between pain and non‐pain events	The use of secondary data Participants had to have an adequate command of English to complete the study ratings

**TABLE 6:** Details/Results extracted from studies that developed and implemented pain management approaches.

SN	Study ID: Author Year Country	Evaluation approach	Most relevant finding(s)	Barrier(s)
10	Graham et al. 2022 Australia	Post‐simulation questionnaire	A relationship between workplace experience and pain recognition was found Although most of the nurses demonstrated adequate formal knowledge of pain, this did not facilitate pain recognition in patients with dementia Insufficient dementia knowledge/training for decision‐making, and/or workplace culture	Contextual factors such as ward busyness and competing pressures on nurses' time were not built into or investigated Nurses' decision‐making was examined only at the individual level, rather than at a collaborative or interprofessional level The authors alluded that, due to the large sample size (*n* = 274), it was impractical to use concurrent verbal‐protocol analysis, which they deemed the best method for gaining introspection into problem‐solvers' appraisal of information
11	Gilmore‐Bykovskyi et al. 2021 USA	Pre‐ and post‐survey data were used to evaluate the perceived feasibility and utility of the intervention tools Exploratory patient outcomes were evaluated through a retrospective chart review of patients who received the PROACTIVE Treatment Approach Review of patients' documentation	The implementation provided insights into opportunities to improve gerontological nursing care for patients and suggested that optimising the physical environment to ingrain person‐centred care delivery tools may be a valuable strategy for adopting similar caregiving approaches in other inpatient settings The authors found it essential to encourage nursing staff autonomy and flexibility in sequencing, using, and completing intervention components to maximise feasibility, given their available time and information resources for the distinct elements	The teaching hospital experienced frequent turnover among interns, residents, attending providers, and nursing staff, which challenged interdisciplinary collaboration Lack of rigorous pre‐ and post‐assessment data regarding behavioural expressions (BE)
12	Lukas et al. 2022 Germany	Before and after comparison Differences between the before‐ and after‐implementation groups were tested using a chi‐squared test or the Fisher exact test for categorical variables, and for normally distributed continuous variables, a *t*‐test for independent samples or the Mann–Whitney *U* test in cases of skewed distributions Linear mixed model analysis was used to assess the potential impact of unequally distributed variables in the before and after cohorts on NPI‐Q‐12 scores	The study showed that introducing and applying an adapted version of STI is feasible in a hospital setting	Nurses who were not trained were also used in each of the four STI application days The 4‐day observation period may not have been long enough to detect significant differences between the two groups regarding BPSD The participants' pain scores may have been too low to expect significant analgesic effects There were no entry criteria, such as a minimum pain score
13	McCorkell et al. 2017 UK	Pre‐implementation and post‐implementation dementia toolkit audits	Education and training were provided to ward staff to ensure the toolkit was implemented appropriately Staff involvement in developing the toolkit	Lack of staff education Small scale: The toolkit was only implemented in one acute trauma and orthopaedics ward
14	Montoro‐Lorite et al. 2020 Spain	The Kolmogorov–Smirnov test was used to calculate the mean, median, mode, minimum, maximum, standard deviation, and 95% confidence interval The Wilcoxon signed‐rank test was used to assess changes in pain development by analysing average scores at admission and discharge across the different scales The Spearman correlation was used to assess the association between score values and the number of nonpharmacological actions in both groups The intraclass correlation coefficient (ICC) was assessed between evaluations using the PAINAD‐Sp and NRS in the IG	There is a need for accurate application and recording of all the protocol stages for comprehensive pain management in people with advanced dementia	All patients were receiving regular analgesics, so the observed pain incidence was low

#### 4.3.2 Most Relevant Findings of Studies

Although complex, implementing an evidence‐based pain assessment tool was considered important in all the studies (Tables 5 and 6). Seven studies reported that pain assessment tools, such as PAINAD, were valid and reliable in effectively assessing and managing pain in patients with dementia (Costardi et al. 2007; Dunford et al. 2022; Ferrari et al. 2009; Montoro‐Lorite et al. 2020; Oosterman et al. 2014; Sampson et al. 2015; van de Rijt et al. 2019) and that the ability to complete a pain assessment does not necessarily imply reliability (Pautex et al. 2005). One study found that introducing innovative pain management approaches, such as STI, is feasible in an acute care setting (Lukas et al. 2022). A relationship between workplace experience and pain management was proven in another study (Graham et al. 2022). Insufficient knowledge and training were noted to be a limitation to implementing an intervention (Graham et al. 2022). Training was required for an intervention to be implemented successfully (Costardi et al. 2007; Ferrari et al. 2009; Fry et al. 2018; Gilmore‐Bykovskyi et al. 2021; McCorkell et al. 2017; Montoro‐Lorite et al. 2020). The level of dementia did not affect the assessment of pain (Natavio et al. 2020), and older age was associated with an increased prevalence of pain (Sampson et al. 2015).

#### 4.3.3 Barriers to the Implementation of Intervention

Several challenges encountered during the implementation of the interventions were cited (Tables 5 and 6). Key among these barriers was the small sample size (Costardi et al. 2007; Ferrari et al. 2009; Fry et al. 2018; McCorkell et al. 2017; Natavio et al. 2020; Oosterman et al. 2014; Pautex et al. 2005; van de Rijt et al. 2019). However, in one study, the authors believed that the large sample size they used had a negative outcome on their study (Graham et al. 2022) because it was impractical for them to use concurrent verbal‐protocol (a technique selected as it was thought to be the best method for gaining introspection into problem‐solvers' appraisal of information) for their analysis. The studies identified contextual factors such as staff turnover and ward busyness as barriers (Gilmore‐Bykovskyi et al. 2021; Graham et al. 2022). Issues with data collection, such as missing data (Fry et al. 2018), secondary data (Dunford et al. 2022), and having a single researcher collect all the data (van de Rijt et al. 2019), were identified as barriers to implementation. Limitations in research design, such as the lack of rigour and repeatability associated with action research, quality improvement programmes, and non‐randomised studies, were observed (Gilmore‐Bykovskyi et al. 2021; Lukas et al. 2022; McCorkell et al. 2017; Oosterman et al. 2014). In addition, researcher bias (Oosterman et al. 2014; Sampson et al. 2015) was added to the list of challenges reported by the authors of the included studies. Other barriers included a lack of a more rigorous evaluation approach (Gilmore‐Bykovskyi et al. 2021), insufficient training for some participants, and a shorter observation period (Lukas et al. 2022).

## 5 Discussion

This scoping review identified 14 studies that investigated pain management approaches to assess and manage pain effectively in patients with dementia. To the best of our knowledge, this is the first scoping review to explore the implementation of pain management approaches and gauge the extent to which they effectively manage pain in patients with dementia in an acute care setting. All included studies were published between 2005 and 2022, predominantly in Europe, and were peer‐reviewed.

There are over 35 pain assessment tools that can evaluate pain in patients with dementia (Corbett et al. 2012; Lichtner et al. 2014). Schofield (2018) suggests that, rather than creating new pain assessment tools, the focus should be on validating existing tools and developing innovative methods to enhance pain management for individuals with dementia, given the variety of assessment tools already available. Many of the studies attempted to validate or test the reliability of existing pain assessment tools and further established their validity or reliability. The review found that pain assessment tools such as PAINAD and NOPPAIN can effectively be used to assess pain in patients with dementia. However, the use of these tools alone does not bring about pain reduction in patients. It was noted that pain assessment tools were not associated with shorter time to analgesia administration. Several researchers have recommended the use of observational/behavioural pain assessment tools for people with dementia due to their ability to improve pain recognition and intensity measurement. Nonetheless, use in clinical practice, especially in acute care, remains predominantly poor. This was mainly attributed to the lack of policy recommendations for pain assessment in this vulnerable population. There is a substantial body of national and international guidance on chronic pain assessment and management that, although not specific to people living with dementia, provides transferable principles relevant to dementia care. These guidelines emphasise biopsychosocial assessment, shared decision‐making, and multimodal pain management, which align with the need for holistic, multidisciplinary approaches in the management of people living with dementia (Briggs et al. 2025; Dowell et al. 2022; NICE 2021; Sandbrink et al. 2023). Thus, a person‐centred approach that requires healthcare staff to develop a clear understanding of the background of the patient with dementia, their interests, and personal history should be adopted to improve pain management. There is also a need to conduct repeated studies to build a body of work on the validity and reliability of these pain assessment tools.

The greater emphasis on pain assessment in this review reflects the existing evidence rather than an imbalance in the review itself. Most studies conducted in acute hospital settings prioritise assessment‐related practices such as the use and development of observational pain assessment tools and documentation, while far fewer studies evaluate pain management interventions for people living with dementia (Closs et al. 2016). This pattern is consistent with wider literature showing that challenges in recognising and assessing pain in people living with dementia have received substantially more research attention than the development and evaluation of pain management strategies (Smith et al. 2023). Consequently, the discussion of management approaches is necessarily limited. The lack of robust evidence about the use of pain management interventions in acute care highlights a notable gap and underscores the need for future research in this area.

Conducting an initial assessment to establish the presence of pain using a pain assessment tool is the first step in managing pain (Torvik et al. 2015). Subsequently, a comprehensive, stepwise approach is needed to effectively manage pain. It is, therefore, important to develop innovative pain management approaches to improve pain management in patients with dementia. Five studies (Gilmore‐Bykovskyi et al. 2021; Graham et al. 2022; Lukas et al. 2022; McCorkell et al. 2017; Montoro‐Lorite et al. 2020) reported on the development and implementation of pain management approaches to effectively assess and manage pain in patients with dementia. One study found that introducing innovative pain management approaches, such as Serial Trial Intervention (STI), was feasible in an acute care setting (Lukas et al. 2022). To facilitate its implementation in acute care settings, the authors developed an adapted version of STI. The data from the study failed to show any increase in the use of analgesia but instead showed a remarkable increase in the use of antidepressants and antipsychotic drugs. The authors assumed that the increase in these drugs may be related to the random higher incidence of delirium and cognitive impairment. They also suggested that STI can be used as a tool to raise awareness about the possible causes of behavioural and psychological symptoms of dementia and pain.

In addition, other innovations, such as the Dementia Toolkit (McCorkell et al. 2017), the Comprehensive Pain Management Protocol in Advanced Dementia (Montoro‐Lorite et al. 2020), the Personalised Approach and Targeted Interventions (PROACTIVE) Treatment Approach (Gilmore‐Bykovskyi et al. 2021) and the Innovative Virtual Simulation of a Vignette (Graham et al. 2022), had the potential to assess and manage pain equally in this population. Nurses from the Trauma and Orthopaedics Unit developed the Dementia toolkit after undertaking an audit of the care of 20 patients with dementia and a fractured neck of the femur admitted to that unit. The audit revealed that these patients were not adequately assessed for pain, and analgesics were not regularly or appropriately administered. However, after the toolkit's introduction, patients' pain management improved. The authors alluded that appropriate pain assessment tools were used and that analgesics were prescribed routinely and as required. Although there were some elements of a person‐centred approach (gathering information about the patient from family members and introducing a traffic light communication sheet for patients with dementia), this was not comprehensive.

A team of experts in dementia care developed the Comprehensive Pain Management Protocol in Advanced Dementia based on a comprehensive literature review to improve nurses' knowledge of pain management. The protocol involved pain assessment, planning and action, reassessment, and registration and record‐keeping. This study's main finding illustrated the significance of assisting nurses with algorithms with clearly described steps to achieve appropriate pain management in patients with dementia. Participants were continuously assessed during the study, and this technique significantly improved pain management. However, the authors found that the incidence of pain was low and attributed this to the fact that all patients were already on a regular analgesic regimen. Sampson et al. (2015) indicated that it is more effective to manage a patient's pain when regular analgesia is prescribed rather than relying on a when‐required prescription. The comparison of pain scores at admission and discharge further demonstrated the protocol's effectiveness and usefulness. The pain scores at discharge were low when compared to the pain scores at admission. Moreover, a positive correlation was observed between pain scores and the number of non‐pharmacological interventions. It was confirmed that high pain scores resulted in an increase in non‐pharmacological interventions being planned and utilised to decrease pain. Although the protocol advocated for the use of a validated and reliable scale to assess pain in patients with dementia, it failed to consider the background of the patient (presence of acute pain, history of chronic pain, and usual cognition). Considering the patient's background when assessing pain would have provided a holistic approach.

The PROACTIVE Treatment Approach considered varying triggers of dementia‐related behavioural expressions (BE) and focused primarily on pain. These symptoms, such as aggression and refusal of treatment, make pain management challenging in this population. This treatment approach was developed through a collaboration between nursing leadership and nursing staff of a general medicine inpatient unit and encompasses preconditions, pre‐implementation, implementation, maintenance, and evolution phases. The core components of the treatment approach pivoted on supporting nurses and healthcare staff to actively prevent dementia‐related BE and to provide person‐centred responses to BE, through four integrated components guided by decision support tools. The components include individualised needs assessment, identifying and treating pain, routine use of prevention strategies and symptom‐specific treatment guidance. After implementing the approach, nurses and healthcare staff reported a boost in confidence in managing BE and adopted more nurse‐sensitive care practices. The results indicated that patients in the post‐implementation cohort had a shorter hospital stay and received a higher administration of scheduled and when‐required paracetamol. The study further demonstrated that evidence‐based prevention and management approaches can be implemented in an inpatient unit. Despite the extensive details and significant findings outlined in this study, the University Institutional Review Board determined it was not research. Therefore, a systematic investigation is required to clarify several aspects of the intervention and consider including a person‐centred review of BE triggers.

Herr et al. (2011) established that the management of agitation caused by pain in patients with dementia is challenging, as most of these patients find it difficult to explain the cause of their agitation. It has been proven that the management of pain‐related agitation in patients with dementia has not been sufficiently studied, thus making it difficult to determine the current interventions being used by healthcare staff (Graham et al. 2022). Considering this, an innovative virtual vignette was developed to examine nurses' performance in recognising and treating pain‐related agitation in a patient with dementia. At the end of the study, it was disclosed that most nurses were unable to identify and treat pain‐related agitation in patients with dementia. Moreover, the inability of nurses to assess pain effectively resulted in 88% of them administering antipsychotics as their first line of action in treating pain‐related agitation. Additionally, although a diagnosis of fracture was documented throughout the patient's records, most of the nurses failed to link this to agitation. Interestingly, 98% of nurses demonstrated in‐depth knowledge of pain management, as indicated by their responses to the ‘Pain‐Knowledge Questionnaire’. However, they were unable to identify pain in the simulation. Thus, the authors concluded that having adequate formal knowledge of pain management does not necessarily facilitate pain recognition in patients with dementia. While the study included a form of pain management approach for patients with dementia, the exact procedures and rationale for the management were not specified. Furthermore, the authors examined nurses' decision‐making only at the individual level. It is essential to state that a collaborative approach is required to manage pain in patients with dementia effectively. This review has revealed a lack of standardised, person‐centred protocols for managing pain in patients with dementia. Therefore, a sequential standardised algorithm with a comprehensive person‐centred pain management approach is needed.

The most dominant strategy identified across the included studies for successful intervention implementation was training. The studies acknowledge that strategies, including training, in‐service meetings, and clinical supervision, were essential to implementing an intervention. They further identified that insufficient knowledge of dementia and inadequate training hindered the implementation process; thus, education and training were required to successfully implement an intervention. It is imperative that these training sessions are organised and delivered in clinical settings to enhance healthcare staff's practice.

Most of the barriers to the implementation were centred on sample size. Determining the sample size is a critical aspect of any study, and it is widely accepted that an appropriate sample size makes the study more efficient, as the data generated tend to be more reliable (Faber and Fonseca 2014). All studies included sample size estimation, and the sample sizes ranged from 20 to 274, depending on the target population and the aim of the study. Many included studies stipulated that their findings could not be generalised because the sample size was small (Costardi et al. 2007; Ferrari et al. 2009; Fry et al. 2018; McCorkell et al. 2017; Natavio et al. 2020; Oosterman et al. 2014; Pautex et al. 2005; van de Rijt et al. 2019). The reasons for these studies' small sample sizes were not clearly documented. However, this could largely be attributed to the study population at the time, the number of participants who met the inclusion criteria or consented to the study, or frequent staff turnover. In a few studies, the authors noted that the small sample size could have affected their statistical analysis and limited the generalisability of the results to the entire population. On the contrary, one study (Graham et al. 2022) listed a large sample size as a barrier. It further explained that, due to the large sample size, it was impractical to use concurrent verbal‐protocol analysis, which they deemed the best method for gaining insight into problem‐solvers' appraisal of information. Researcher bias, contextual factors and issues with data collection accounted for additional barriers in the studies. Missing data in small numbers of patients, which may have resulted from competing nurse workloads, could have influenced study findings. Also, contextual factors, such as ward busyness and time constraints, could affect care decisions for patients with dementia. Moreover, studies in hospitals with frequent staff turnover could hinder interdisciplinary collaboration.

Only three studies (Gilmore‐Bykovskyi et al. 2021; Graham et al. 2022; Natavio et al. 2020) stated that conceptual frameworks guided the implementation of their intervention. Theoretical frameworks explain and interpret the phenomenon under study, while conceptual frameworks clarify assumptions about it (Luft et al. 2022). Despite the importance of their constructs in research, the use of theoretical and conceptual frameworks is relatively obscure in the vast amount of available literature (Green 2014). The use of conceptual frameworks significantly guided these three studies in various ways. This included helping to examine real‐time clinical decision‐making in medical/surgical nurses, serving as a roadmap for adopting and sustaining interventions in real‐world settings, and increasing the focus on comparisons between and within two selected observational tools for patients undergoing a standardised pain stimulus. Nonetheless, the remaining eleven studies successfully carried out their research without applying a conceptual framework. They, however, acknowledge the need for a more rigorous evaluation approach to determine the success or otherwise of an intervention. Interestingly, none of the included studies employed an evaluative framework to ascertain whether the intervention was successful.

### 5.1 Strengths and Limitations

The review has several strengths. The most important strength was the limitation of selection bias. Numerous steps were taken to limit selection bias, and the four authors independently reviewed the studies. In addition, the JBI guidelines and a priori registered protocol were used to conduct this scoping review. The first author, in collaboration with an experienced subject librarian, developed a comprehensive search strategy. There were no restrictions on publication or study design.

Nonetheless, this review has some limitations that need to be considered. Given the authors' efforts to eliminate publication bias through a comprehensive search strategy, a pragmatic decision was made to include only English‐language publications during screening. This resulted in the exclusion of 11 studies from non‐English countries from the initial screening. It is also important to note that, like any review, this scoping review's findings depend on the information provided in the publications analysed. Hence, there is a possibility of publication bias in the source literature. Finally, the review only considered publications from 2005; as a result, studies published before 2005 were not captured.

## 6 Conclusion

This review has revealed a lack of standardised, person‐centred approaches to pain management for people with dementia in acute care settings. Moreover, there is a dearth of evidence demonstrating the effectiveness of these approaches in achieving pain reduction within this client group. While valid pain assessment tools exist, their use alone does not guarantee timely or effective pain relief. This review highlights that addressing pain in people with dementia requires a comprehensive, person‐centred management approach that goes beyond assessment to include timely interventions and ongoing evaluation. Education on pain management is vital; however, it alone is not sufficient to lead to improvements in care. Limitations were reported in the study designs, including a small sample size and few patient‐centred outcomes. Barriers to the implementation of research were noted, including the impact of contextual factors such as time constraints and the busyness of the wards, and there appears to be a lack of policy regarding the use of pain assessment tools and management of pain among these vulnerable patients.

## 7 Implications for Practice

The review identified several validated instruments for assessing pain in people living with dementia; however, the availability of tools alone is insufficient to change practice. Effective pain management requires that assessment be embedded within a broader, systematic approach that leads directly to timely and appropriate management. Nurses, therefore, need managerial support to integrate observational pain assessment tools into routine workflows, link assessments to clear clinical actions and avoid diagnostic overshadowing by recognising pain as a potential cause of behavioural change. Ongoing education and periodic training in dementia‐specific pain assessment and management are essential to strengthen nurses' confidence and competence. Management of acute healthcare facilities must go beyond delivering routine training or periodic updates to nursing. Sustained managerial support is required to promote the consistent use of evidence‐based practices and to cultivate a clinical culture in which effective pain management is recognised as a core priority. This involves providing ongoing mentorship, reinforcing the importance of pain management, ensuring access to appropriate pain assessment tools and resources, and creating an environment where nurses feel empowered and expected to advocate for optimal pain relief for all patients, including those living with dementia. Additionally, incorporating insights from family and carers, alongside improved documentation and communication within the multidisciplinary team, can enhance person‐centred, safe pain management in acute care settings.

The review further highlights the need for repeated, rigorous studies to build a stronger body of evidence on the validity, reliability, and real‐world utility of pain assessment tools in acute care. Future research should also extend beyond assessment to evaluate the effectiveness of pharmacological and non‐pharmacological pain management strategies for people living with dementia in hospital settings. Understanding organisational barriers and facilitators is critical for designing interventions that can be successfully implemented and sustained. By outlining implementation strategies and identifying common barriers, this review provides insights to the global clinical community. It provides a foundation for developing and evaluating comprehensive pain management approaches tailored to this population.

## Author Contributions

F.M.: Proposed the concept of conducting a scoping review, developed the protocol for the study, carried out the various stages outlined in the protocol, and drafted the manuscript. D.B., D.H. and V.C.: Supervised, reviewed and edited the manuscript. All authors have read and agreed to the published version of the manuscript.

## Funding

This study was completed through a PhD studentship awarded to the first author by the Ulster University Vice‐Chancellor Research Scholarship.

## Ethics Statement

The authors have nothing to report.

## Consent

The authors have nothing to report.

## Conflicts of Interest

The authors declare no conflicts of interest.

## Supporting information


**Table S1:** EQUATOR Checklist (PRISMA‐ScR).

**Figure S2:** Steps and details of search terms used.

## Data Availability

The data that support the findings of this study are presented in the article and available in the Supporting Information of this article.

## References

[nop270529-bib-0001] Achterberg, W. P. , A. Erdal , B. S. Husebo , M. Kunz , and S. Lautenbacher . 2021. “Are Chronic Pain Patients With Dementia Being Undermedicated?” Journal of Pain Research 14: 431–439. 33623425 10.2147/JPR.S239321 PMC7894836

[nop270529-bib-0002] Ahn, H. , C. Garvan , and D. Lyon . 2015. “Pain and Aggression in a Nursing Home Residents With Dementia: Minimum Data Set 3.0 Analysis.” Nursing Research 64: 256–263. 26126060 10.1097/NNR.0000000000000099

[nop270529-bib-0003] Arksey, H. , and L. O'Malley . 2005. “Scoping Studies: Towards a Methodological Framework.” International Journal of Social Research Methodology 8, no. 1: 19–32.

[nop270529-bib-0005] Bonser, S. J. 2025. “Challenges of Assessing Pain in People With Dementia: A Systematised Literature Review.” Nursing Older People 37, no. 6: 28–33. 40836828 10.7748/nop.2025.e1518

[nop270529-bib-0006] Boulton, R. , J. Sandall , and N. Sevdalis . 2020. “The Cultural Politics of ‘Implementation Science’.” Journal of Medical Humanities 41: 379–394. 31965463 10.1007/s10912-020-09607-9 PMC7343725

[nop270529-bib-0007] Bradbury‐Jones, C. , H. Aveyard , O. R. Herber , L. Isham , J. Taylor , and L. O'Malley . 2021. “Scoping Reviews: The PAGER Framework for Improving the Quality of Reporting.” International Journal of Social Research Methodology 25: 1–14.

[nop270529-bib-0008] Briggs, A. M. , N. Siegfried , R. Waller , et al. 2025. “Clinical Practice Guidelines for the Care of People Experiencing Chronic Primary Pain: Protocol for a Systematic Review With Interpretation Against an Established Chronic Pain Care Priority Framework.” BMJ Open 15, no. 9: e105315. 10.1136/bmjopen-2025-105315 PMC12458848 40973368

[nop270529-bib-0009] Brown, D. , and B. G. McCormack . 2011. “Developing the Practice Context to Enable More Effective Pain Management With Older People: An Action Research Approach.” Implementation Science: IS 6: 9. 21284857 10.1186/1748-5908-6-9 PMC3037913

[nop270529-bib-0010] Bullock, L. , J. Bedson , J. L. Jordan , B. Bartlam , C. A. Chew‐Graham , and P. Campbell . 2019. “Pain Assessment and Pain Treatment for Community‐Dwelling People With Dementia: A Systematic Review and Narrative Synthesis.” International Journal of Geriatric Psychiatry 34, no. 6: 807–821. 30724409 10.1002/gps.5078

[nop270529-bib-0011] Bullock, L. , C. A. Chew‐Graham , J. Bedson , B. Bartlam , and P. Campbell . 2020. “The Challenge of Pain Identification, Assessment, and Management in People With Dementia: A Qualitative Study.” BJGP Open 4, no. 2: bjgpopen20X101040. 10.3399/bjgpopen20X101040 PMC7330220 32457099

[nop270529-bib-0012] Claridge, J. A. , and T. C. Fabian . 2005. “History and Development of Evidence‐Based Medicine.” World Journal of Surgery 29, no. 5: 547–553. 15827845 10.1007/s00268-005-7910-1

[nop270529-bib-0013] Closs, S. J. , S. Closs , D. Dowding , et al. 2016. “Towards Improved Decision Support in the Assessment and Management of Pain for People With Dementia in Hospital: A Systematic Meta‐Review and Observational Study.” Health and Social Care Delivery Research 4, no. 30: 1–162. 27786433

[nop270529-bib-0014] Cook, S. C. , A. C. Schwartz , and N. J. Kaslow . 2017. “Evidence‐Based Psychotherapy: Advantages and Challenges.” Neurotherapeutics 14: 537–545. 28653278 10.1007/s13311-017-0549-4 PMC5509639

[nop270529-bib-0015] Corbett, A. , B. Husebo , M. Malcangio , et al. 2012. “Assessment and Treatment of Pain in People With Dementia.” Nature Reviews Neurology 8, no. 5: 264–274. 22487749 10.1038/nrneurol.2012.53

[nop270529-bib-0017] Costardi, D. , L. Rozzini , C. Costanzi , et al. 2007. “The Italian Version of the Pain Assessment in Advanced Dementia (PAINAD) Scale.” Archives of Gerontology and Geriatrics 44, no. 2: 175–180. 16730814 10.1016/j.archger.2006.04.008

[nop270529-bib-0018] Covidence Systematic Review Software . 2024. “Veritas Health Innovation, Melbourne, Australia.” http://www.covidence.org.

[nop270529-bib-0019] Dowell, D. , K. R. Ragan , C. M. Jones , G. T. Baldwin , and R. Chou . 2022. “CDC Clinical Practice Guideline for Prescribing Opioids for Pain—United States, 2022.” MMWR—Recommendations and Reports 71, no. 3: 1–95. 10.15585/mmwr.rr7103a1 PMC9639433 36327391

[nop270529-bib-0020] Dunford, E. , E. West , and E. L. Sampson . 2022. “Psychometric Evaluation of the Pain Assessment in Advanced Dementia Scale in an Acute General Hospital Setting.” International Journal of Geriatric Psychiatry 37, no. 12: 1–10. 10.1002/gps.5830. PMC9828226 36317464

[nop270529-bib-0021] Faber, J. , and L. M. Fonseca . 2014. “How Sample Size Influences Research Outcomes.” Dental Press Journal of Orthodontics 19, no. 4: 27–29. 10.1590/2176-9451.19.4.027-029.ebo PMC4296634 25279518

[nop270529-bib-0022] Ferrari, R. , M. Martini , S. Mondini , et al. 2009. “Pain Assessment in Non‐Communicative Patients: The Italian Version of the Non‐Communicative Patient's Pain Assessment Instrument (NOPPAIN).” Aging Clinical and Experimental Research 21, no. 4–5: 298–306. 19959918 10.1007/BF03324919

[nop270529-bib-0023] Fry, M. , L. Chenoweth , and G. Arendts . 2018. “Can an Observational Pain Assessment Tool Improve Time to Analgesia for Cognitively Impaired Older Persons? A Cluster Randomised Controlled Trial.” Emergency Medicine Journal 35, no. 1: 33–38. 28780493 10.1136/emermed-2016-206065

[nop270529-bib-0024] Gilmore‐Bykovskyi, A. , M. Markart , K. Imig , et al. 2021. “Implementation and Evaluation of an Acute Care Multicomponent Intervention for Dementia‐Related Behavioural Expressions.” Journal of Gerontological Nursing 47, no. 9: 21–30. 10.3928/00989134-20210803-02 PMC8422803 34432573

[nop270529-bib-0025] Global Burden of Disease (GBD) 2019 Dementia Forecasting Collaborators . 2022. “Estimation of the Global Prevalence of Dementia in 2019 and Forecasted Prevalence in 2050: An Analysis for the Global Burden of Disease Study 2019.” Lancet. Public Health 7, no. 2: e105–e125. 34998485 10.1016/S2468-2667(21)00249-8 PMC8810394

[nop270529-bib-0026] Graham, F. , E. Beattie , and E. Fielding . 2022. “Hospital Nurses' Management of Agitation in Older Cognitively Impaired Patients: Do They Recognise Pain‐Related Agitation?” Age and Ageing 51, no. 7: afac140. 35796135 10.1093/ageing/afac140

[nop270529-bib-0027] Green, H. E. 2014. “Use of Theoretical and Conceptual Frameworks in Qualitative Research.” Nurse Researcher 21, no. 6: 34–38. 10.7748/nr.21.6.34.e1252 25059086

[nop270529-bib-0028] Harkin, D. , V. Coates , and D. Brown . 2022. “Exploring Ways to Enhance Pain Management for Older People With Dementia in Acute Care Settings Using a Participatory Action Research Approach.” International Journal of Older People Nursing 17, no. 6: e12487. 35761509 10.1111/opn.12487 PMC9787744

[nop270529-bib-0029] Harmon, J. , P. Summons , and I. Higgins . 2019. “Experiences of the Older Hospitalised Person on Nursing Pain Care: An Ethnographic Insight.” Journal of Clinical Nursing 28, no. 23–24: 4447–4459. 31408553 10.1111/jocn.15029

[nop270529-bib-0030] Herr, K. , P. J. Coyne , M. McCaffery , R. Manworren , and S. Merkel . 2011. “Pain Assessment in the Patient Unable to Self‐Report: Position Statement With Clinical Practice Recommendations.” Pain Management Nursing 12: 230–250. 22117755 10.1016/j.pmn.2011.10.002

[nop270529-bib-0031] Hoffmann, F. , H. van den Bussche , B. Wiese , G. Glaeske , and H. Kaduszkiewicz . 2014. “Diagnoses Indicating Pain and Analgesic Drug Prescription in Patients With Dementia: A Comparison to Age‐ and Sex‐Matched Controls.” BMC Geriatrics 14, no. 1: 20. 24520876 10.1186/1471-2318-14-20 PMC3937236

[nop270529-bib-0032] Ingelson, B. , S. Dahlke , H. O'Rourke , and G. Low . 2024. “A Scoping Review on Nurse's Pain Management of Older Patients With Dementia in a Hospital Environment.” Pain Management Nursing: Official Journal of the American Society of Pain Management Nurses 25, no. 2: 104–112. 37968142 10.1016/j.pmn.2023.10.004

[nop270529-bib-0033] Jonsdottir, T. , and E. C. Gunnarsson . 2021. “Understanding Nurses' Knowledge and Attitudes Toward Pain Assessment in Dementia: A Literature Review.” Pain Management Nursing: Official Journal of the American Society of Pain Management Nurses 22, no. 3: 281–292. 33334680 10.1016/j.pmn.2020.11.002

[nop270529-bib-0034] Liao, Y. J. , Y. L. Jao , D. Berish , et al. 2023. “A Systematic Review of Barriers and Facilitators of Pain Management in Persons With Dementia.” Journal of Pain 24, no. 5: 730–741. 36634886 10.1016/j.jpain.2022.12.014

[nop270529-bib-0035] Lichtner, V. , D. Dowding , N. Allcock , et al. 2016. “The Assessment and Management of Pain in Patients With Dementia in Hospital Settings: A Multi‐Case Exploratory Study From a Decision‐Making Perspective.” BMC Health Services Research 16, no. 1: 427. 27553364 10.1186/s12913-016-1690-1 PMC4995653

[nop270529-bib-0036] Lichtner, V. , D. Dowding , P. Esterhuizen , et al. 2014. “Pain Assessment for People With Dementia: A Systematic Review of Systematic Reviews of Pain Assessment Tools.” BMC Geriatrics 14: 138. 25519741 10.1186/1471-2318-14-138 PMC4289543

[nop270529-bib-0037] Luft, J. A. , S. Jeong , R. Idsardi , and G. Gardner . 2022. “Literature Reviews, Theoretical Frameworks, and Conceptual Frameworks: An Introduction for New Biology Education Researchers.” CBE Life Sciences Education 21, no. 3: rm33. 35759629 10.1187/cbe.21-05-0134 PMC9582830

[nop270529-bib-0038] Lukas, A. , M. Bienas , B. Mayer , L. Radbruch , and I. Gnass . 2022. “Responsive Behaviours and Pain Management in Hospital Dementia Care: A Before and After Comparison of the ‘Serial Trial Intervention’.” Frontiers in Pain Research 3: 810804. 35599966 10.3389/fpain.2022.810804 PMC9121813

[nop270529-bib-0039] Mattia, A. , and A. Blasimme . 2024. “From Research to Policy: Unveiling Dementia Prevention Efforts in Switzerland.” Journal of Aging & Social Policy 38, no. 1: 16–36. 38179785 10.1080/08959420.2023.2297602

[nop270529-bib-0040] McCorkell, G. , D. Harkin , V. McCrory , M. Lafferty , and V. Coates . 2017. “Care of Patients With Dementia in an Acute Trauma and Orthopaedics Unit.” Nursing Standard 31, no. 36: 44–53. 10.7748/ns.2017.e10250 28466701

[nop270529-bib-0041] Montoro‐Lorite, M. , E. Risco , M. Canalias‐Reverter , J. A. Rodríguez‐Murillo , M. García‐Pascual , and A. Zabalegui . 2020. “Integrated Management of Pain in Advanced Dementia.” Pain Management Nursing 21, no. 4: 331–338. 32253093 10.1016/j.pmn.2019.12.004

[nop270529-bib-0042] Morrison, R. S. , and A. L. Siu . 2000. “A Comparison of Pain and Its Treatment in Advanced Dementia and Cognitively Intact Patients With Hip Fracture.” Journal of Pain and Symptom Management 19, no. 4: 240–248. 10799790 10.1016/s0885-3924(00)00113-5

[nop270529-bib-0043] Natavio, T. , E. McQuillen , M. S. Dietrich , et al. 2020. “A Comparison of the Pain Assessment Checklist for Seniors With Limited Ability to Communicate (PACSLAC) and Pain Assessment in Advanced Dementia Scale (PAINAD).” Pain Management Nursing 21, no. 6: 502–509. 32475696 10.1016/j.pmn.2020.04.001

[nop270529-bib-0044] National Institute for Health and Care Excellence . 2021. Chronic Pain (Primary and Secondary) in Over 16s: Assessment of All Chronic Pain and Management of Chronic Primary Pain (NICE Guideline NG193). NICE. 33939353

[nop270529-bib-0045] Oosterman, J. M. , H. Hendriks , S. Scott , K. Lord , N. White , and E. L. Sampson . 2014. “When Pain Memories Are Lost: A Pilot Study of Semantic Knowledge of Pain in Dementia.” Pain Medicine 15, no. 5: 751–757. 24401151 10.1111/pme.12336

[nop270529-bib-0046] Page, M. J. , J. E. McKenzie , P. M. Bossuyt , et al. 2021. “The PRISMA 2020 Statement: An Updated Guideline for Reporting Systematic Reviews.” BMJ (Clinical Research Ed.) 372: n71. 10.1136/bmj.n71 PMC8005924 33782057

[nop270529-bib-0047] Pautex, S. , F. Herrmann , P. Le Lous , M. Fabjan , J. P. Michel , and G. Gold . 2005. “Feasibility and Reliability of Four Pain Self‐Assessment Scales and Correlation With an Observational Rating Scale in Hospitalised Elderly Demented Patients.” Journals of Gerontology. Series A, Biological Sciences and Medical Sciences 60, no. 4: 524–529. 15933396 10.1093/gerona/60.4.524

[nop270529-bib-0048] Peisah, C. , J. Weaver , L. Wong , and J. A. Strukovski . 2014. “Silent and Suffering: A Pilot Study Exploring Gaps Between Theory and Practice in Pain Management for People With Severe Dementia in Residential Aged Care Facilities.” Clinical Interventions in Aging 9: 1767–1774. 25342895 10.2147/CIA.S64598 PMC4205115

[nop270529-bib-0049] Peters, M. D. J. , C. Marnie , A. C. Tricco , et al. 2020. “Updated Methodological Guidance for the Conduct of Scoping Reviews.” JBI Evidence Synthesis 18, no. 10: 2119–2126. 33038124 10.11124/JBIES-20-00167

[nop270529-bib-0050] Pieper, M. J. , A. H. van Dalen‐Kok , A. L. Francke , et al. 2013. “Interventions Targeting Pain or Behaviour in Dementia: A Systematic Review.” Ageing Research Reviews 12, no. 4: 1042–1055. 23727161 10.1016/j.arr.2013.05.002

[nop270529-bib-0051] Pu, L. , M. Barton , M. Kodagoda Gamage , M. Okada , M. Todorovic , and W. Moyle . 2025. “Pain Assessment and Management in Dementia Care: Qualitative Perspectives of People With Dementia, Their Families, and Healthcare Professionals.” Journal of Clinical Nursing 34, no. 7: 2933–2947. 40200556 10.1111/jocn.17771 PMC12181161

[nop270529-bib-0052] Resnick, B. , M. Boltz , E. Galik , et al. 2023. “A Descriptive Study of Treatment of Pain in Acute Care for Patients Living With Dementia.” Pain Management Nursing 24, no. 3: 248–253. 36737349 10.1016/j.pmn.2022.12.010 PMC10213108

[nop270529-bib-0053] Sampson, E. L. , N. White , K. Lord , et al. 2015. “Pain, Agitation, and Behavioural Problems in People With Dementia Admitted to General Hospital Wards: A Longitudinal Cohort Study.” Pain 156, no. 4: 675–683. 25790457 10.1097/j.pain.0000000000000095 PMC4381983

[nop270529-bib-0054] Sandbrink, F. , J. L. Murphy , M. Johansson , et al. 2023. “The Use of Opioids in the Management of Chronic Pain: Synopsis of the 2022 Updated U.S. Department of Veterans Affairs and U.S. Department of Defense Clinical Practice Guideline.” Annals of Internal Medicine 176, no. 3: 388–397. 36780654 10.7326/M22-2917

[nop270529-bib-0055] Schofield, P. 2018. “The Assessment of Pain in Older People: UK National Guidelines.” Age and Ageing 47, no. suppl_1: i1–i22. 29579142 10.1093/ageing/afx192 PMC5888957

[nop270529-bib-0056] Smith, T. O. , D. Lockey , H. Johnson , L. Rice , J. Heard , and L. Irving . 2023. “Pain Management for People With Dementia: A Cross‐Setting Systematic Review and Meta‐Ethnography.” British Journal of Pain 17, no. 1: 6–22. 36815066 10.1177/20494637221119588 PMC9940246

[nop270529-bib-0057] Torvik, K. , B. Nordtug , I. K. Brenne , and M. Rognstad . 2015. “Pain Assessment Strategies in Home Care and Nursing Homes in Mid‐Norway: A Cross‐Sectional Survey.” Pain Management Nursing 16, no. 4: 602–608. 25982750 10.1016/j.pmn.2015.01.001

[nop270529-bib-0058] Tricco, A. C. , E. Lillie , W. Zarin , et al. 2018. “PRISMA Extension for Scoping Reviews (PRISMA‐ScR): Checklist and Explanation.” Annals of Internal Medicine 169, no. 7: 467–473. 30178033 10.7326/M18-0850

[nop270529-bib-0059] van de Rijt, L. J. , R. A. Weijenberg , A. R. Feast , et al. 2019. “Orofacial Pain During Rest and Chewing in Dementia Patients Admitted to Acute Hospital Wards: Validity Testing of the Orofacial Pain Scale for Non‐Verbal Individuals.” Journal of Oral & Facial Pain and Headache 33, no. 3: 247–253. 30304081 10.11607/ofph.2136

[nop270529-bib-0060] van Kooten, J. , S. Delwel , T. T. Binnekade , et al. 2015. “Pain in Dementia: Prevalence and Associated Factors: Protocol of a Multidisciplinary Study.” BMC Geriatrics 15, no. 1: 29. 25879681 10.1186/s12877-015-0025-0 PMC4436741

[nop270529-bib-0061] Wideman, T. H. , R. R. Edwards , D. M. Walton , M. O. Martel , A. Hudon , and D. A. Seminowicz . 2019. “The Multimodal Assessment Model of Pain: A Novel Framework for Further Integrating the Subjective Pain Experience Within Research and Practice.” Clinical Journal of Pain 35, no. 3: 212–221. 30444733 10.1097/AJP.0000000000000670 PMC6382036

[nop270529-bib-0062] World Health Organization . 2014. Ghana Country Assessment Report on Ageing and Health. WHO Press.

[nop270529-bib-0063] World Health Organization . 2023. “World Health Organisation.” https://www.who.int/news‐room/fact‐sheets/detail/dementia.

