# Supplementary material for: Implementing Pain Management Approaches Among Patients With Dementia in an Acute Hospital Setting: A Scoping Review
Source: Nurs Open. 2026 Apr 7;13(4):e70529. doi: 10.1002/nop2.70529 (PMC13056697; doi:10.1002/nop2.70529)
Supplement: Supplementary file 2 — Figure S2: Steps and details of search terms used. [file NOP2-13-e70529-s002.docx]

**Steps and details of search terms used**
